# Hyper-replicative bovine leukemia virus by mutation of an envelope N-linked glycosylation site

**DOI:** 10.1186/1742-4690-11-S1-P141

**Published:** 2014-01-07

**Authors:** A de Brogniez, AB Bouzar, J-R Jacques, N Gillet, O Pritsch, L Tomé, M Reichert, L Willems

**Affiliations:** 1 GxABT and GIGA, University of Liege, Gembloux and Liège, Belgium; 2 Department of Pathology, National Veterinary Research Institute, Pulawy, Poland; 3 Protein Biophysic Unit, Pasteur Institute, Montevideo, Uruguay

## 

Reverse genetics can be used in the bovine leukemia virus (BLV) system to characterize mechanisms of viral persistence and pathogenesis. The question addressed here pertains to the role of glycans bound to the BLV envelope glycoprotein (SU). A commonly accepted hypothesis is that addition of carbohydrates to the SU protein potentially creates a structure called « glycan shield » that confers resistance to the virus against the host immune response. On the other hand, glycosylation can also modulate attachment of the virus to the cell membrane. To unravel the role of SU glycosylation, three complementary strategies were developed: pharmacological inhibition of different glycosylation pathways, interference with glycan attachment and site-directed mutagenesis of N-glycosylation sites in an infectious BLV provirus. The different approaches show that glycosylation is required for cell fusion, as expected. Simultaneous mutation of all 8 potential N-glycosylation sites destroys infectivity. Surprisingly, mutation of the asparagine residue at position 230 creates a virus having an increased capacity to form syncytia in vitro. Compared to wild-type BLV, mutant N230 also replicates at accelerated rates in vivo. Collectively, this data thus illustrates an example of a N-glycosylation site that restricts viral replication, contrasting with the hypothesis supported by glycan shield model.

